# Supplementary material for: Phase angle is independently associated with muscle strength across multiple handgrip strength metrics in young adults: A cross-sectional study
Source: PLoS One. 2026 Jun 1;21(6):e0350460. doi: 10.1371/journal.pone.0350460 (PMC13225386; doi:10.1371/journal.pone.0350460)
Supplement: S1 File — The overall correlation is shown for descriptive purposes and is partly driven by between-sex differences). Supplementary Figure S2. Phase angle vs physical activity levels (overall trend P < 0.001). Supplementary Figure S3. Sensitivity analysis for phase angle by diseases status. Supplementary Figure S4. Exploratory ROC analysis of phase angle for low HGS defined by sex-specific tertiles. Supplementary Figure S5. Sampling distribution by academic field, sex composition, phase angle variation, and phase angle–HGS association (n = 1,125). Supplementary Table S1. Low muscle strength was defined using sex- and age-specific international normative values below the 10th percentile for HGS. Supplementary Table S2. Sensitivity analysis for phase angle–HGS association across self-reported disease status. Supplementary Table S3. Sensitivity analysis for phase angle–HGS association and physical activity levels (IPAQ). (PDF) [file pone.0350460.s001.pdf]

Supplementary Files 1

Phase Angle Is Independently Associated with Muscle Strength Across Multiple Handgrip Strength Metrics in Young Adults: A Cross-Sectional Study

Juan Carlos Calderón-González[1], Luis Hebert Palma-Pulido[1], Gonzalo Romero-Martínez[1], Juan Carlos Urriago-Fontal[1], María Elisa Álvarez-Ossa[1], Frank Carrera-Gil[2], Robinson Ramírez-Vélez[1,3,4*]

1. Facultad de Ciencias de la Educación, Unidad Central del Valle del Cauca (UCEVA), Tuluá, Colombia.

2. Department of Food and Nutrition, Faculty of Health Sciences, Pontificia Universidad Javeriana Seccional Cali, Cali 760021, Colombia.

3. Navarrabiomed, Hospital Universitario de Navarra (HUN), Universidad Pública de Navarra (UPNA), Instituto de Investigación Sanitaria de Navarra (IdiSNA), Pamplona, España.

4. CIBER of Frailty and Healthy Aging (CIBERFES), Instituto de Salud Carlos III, Madrid, Spain.

***Corresponding author:** Robinson Ramirez-Velez, PhD, E-mail address: robin640@hotmail.com // robinson.ramirez@unavarra.es

Supplementary Figure S1. Phase angle vs HGS by sex (overall ρ=0.657, P<0.001. The overall correlation is shown for descriptive purposes and is partly driven by between-sex differences.)

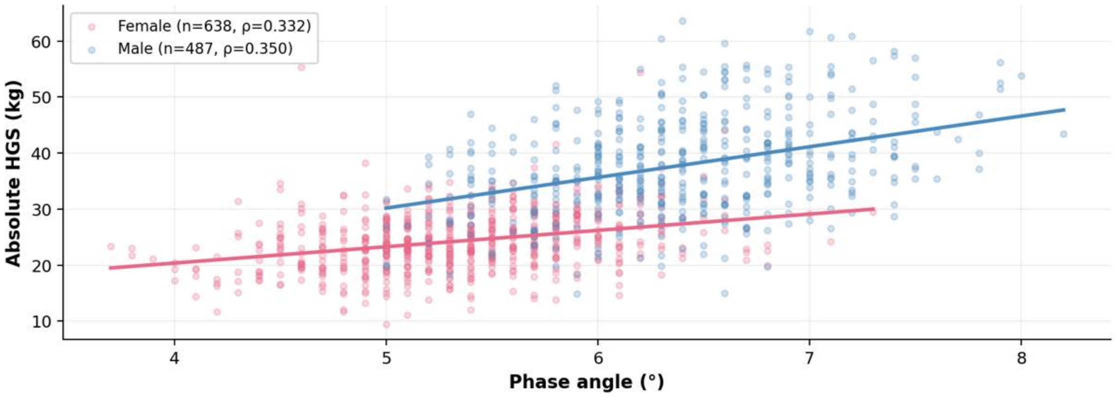

Supplementary Figure S2. Phase angle vs physical activity levels (overall trend P<0.001)

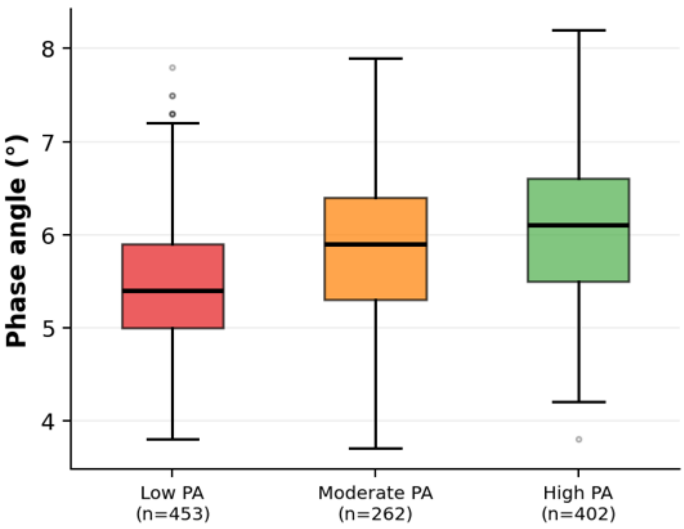

Supplementary Figure S3. Sensitivity analysis for phase angle by diseases status

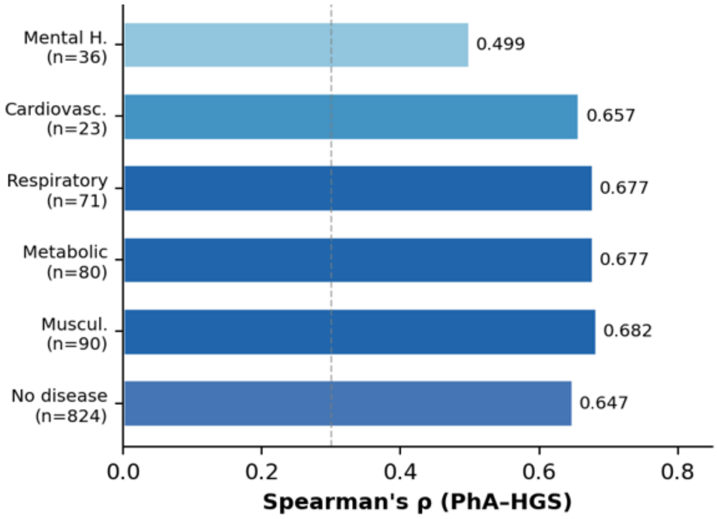

Supplementary Figure S4. Exploratory ROC analysis of phase angle for low HGS defined by sex-specific tertiles

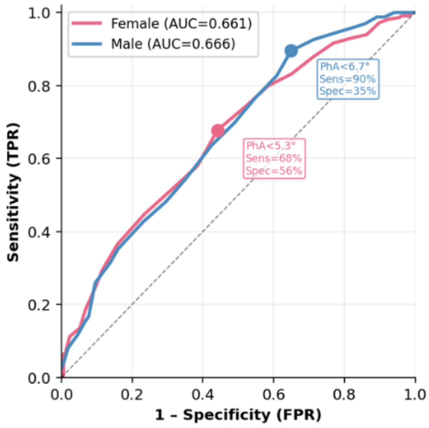

	Female	Male
Low HGS definition (T33)	≤22.1 kg	≤33.4 kg
Low HGS n	214/638	162/487
**AUC**	**0.661**	**0.666**
**Optimal PhA cut-off (Youden index)**	**≤5.30°**	**≤6.70°**
Sensitivity	67.8%	89.5%
Specificity	55.9%	35.1%
Youden index (J)	0.237	0.246

T33 cutoffs were sample-derived tertiles; P10 cutoffs were external international normative values.

Supplementary Table S1. Low muscle strength was defined using sex- and age-specific international normative values below the 10th percentile for HGS

	**Female (HGS=21.7 kg)**	**Male (HGS=36.8 kg)**
AUC	0.651	0.670
Optimal PhA (Youden)	≤5.40°	≤6.30°
Sensitivity	71.0%	62.1%
Specificity	51.7%	63.1%

Supplementary Figure S5. Sampling distribution by academic field, sex composition, phase angle variation, and phase angle–HGS association (n=1,125)

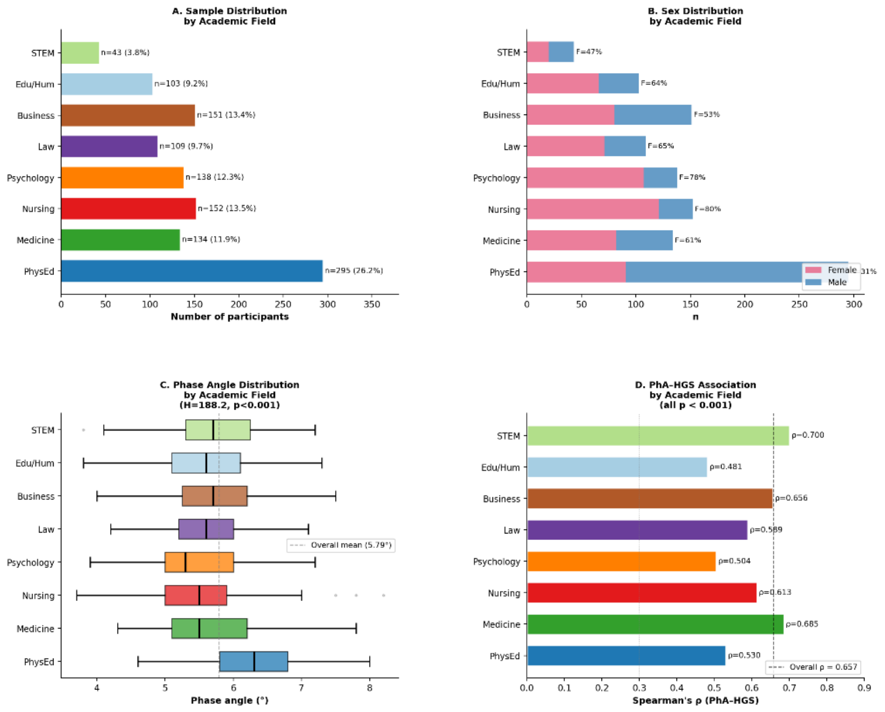

Figure Footnote: Panel A shows the distribution of participants across academic fields. Panel B illustrates sex distribution within each field. Panel C displays phase angle distributions by academic field (overall mean, 5.79°), with between-group differences (H = 188.2; p < 0.001). Panel D presents field-specific correlations between phase angle

and handgrip strength, with all associations reaching statistical significance ($p < 0.001$). The dashed line indicates the overall correlation ($\rho = 0.657$). Variability in sample composition across academic fields should be considered when interpreting subgroup estimates and comparative effect sizes.

Supplementary Table S2. Sensitivity analysis for phase angle–HGS association across self-reported disease subgroups

**Subgroup**	**n**	**ρ (PhA–HGS)**	**p**
No disease (healthy)	824	0.647	<0.001
Musculoskeletal disease	90	0.682	<0.001
Metabolic or obesity	80	0.677	<0.001
Respiratory disease	71	0.677	<0.001
Cardiovascular disease	23	0.657	<0.001
Mental health conditions	36	0.499	0.002

Participants could report more than one disease category; therefore, subgroup counts are not mutually exclusive.

***Supplementary Table S3*. Sensitivity analysis for phase angle–HGS association and physical activity levels (IPAQ)**

**PA level**	**n**	**ρ (PhA–HS)**	**p**
Low PA	453	0.585	<0.001
Moderate PA	262	0.622	<0.001
High PA	402	0.648	<0.001

IPAQ data were available for 1117 participants; 8 participants had missing PA classification. PA categories were defined according to IPAQ scoring criteria.
