# Supplementary material for: Phase angle is independently associated with muscle strength across multiple handgrip strength metrics in young adults: A cross-sectional study
Source: PLoS One. 2026 Jun 1;21(6):e0350460. doi: 10.1371/journal.pone.0350460 (PMC13225386; doi:10.1371/journal.pone.0350460)
Supplement: Attachment — Submitted filename: ReplyR1.docx [file pone.0350460.s003.docx]

Dear Dr. Nunes and Reviewers,

Ref: **Manuscript ID:** PONE-D-26-07944. **Title:** Phase Angle Is Independently Associated with Muscle Strength Across Multiple Handgrip Strength Metrics in Young Adults: A Cross-Sectional Study

We thank you for your careful evaluation of our manuscript and for the constructive comments provided. We have revised the manuscript extensively to improve methodological transparency, statistical reporting, and clarity of interpretation. All changes have been incorporated into the revised manuscript and are highlighted in the tracked-changes version.

Below, we provide a point-by-point response to each comment.

**Reviewer #1:**

This manuscript investigates the effects of a specific training intervention on performance and physiological responses in athletes. The topic is relevant to the field of sports science. The study appears to follow an applied experimental design, and the topic may have practical implications for training prescription. The manuscript addresses an interesting and relevant question; however, improvements in methodological transparency, statistical reporting, and clarity of interpretation are required before the study can be suitable for publication.
**Response:** *Thank you very much for your positive evaluation of the revised manuscript and for your constructive feedback.*

**Comment 1:** Major Comments- #Study Design and Experimental Control
The manuscript would benefit from a clearer and more detailed description of the experimental design. Please clarify:
-Whether the study followed a randomized design.
-How participants were allocated to experimental conditions.
-Whether any control group or comparison condition was included.
-How potential confounding factors (training load outside the study, fatigue, or prior experience) were controlled.
-Providing a schematic diagram of the study design could significantly improve clarity.
**Response:** *We agree that the study design required clearer description. We have explicitly clarified that this study is a cross-sectional observational analysis with no intervention, randomization, or allocated to experimental conditions or control group. We clarified that the eligibility criteria apply specifically to the present cross-sectional analysis within the EpiHealth-UCEVA project and explicitly reported the data collection period in the Methods section as: “This study used a cross-sectional observational design.”*

**Comment 2:** #Sample Size and Statistical Power
The manuscript does not clearly report whether an a priori power analysis was conducted. Please clarify:
-The expected effect size used to estimate the sample size.
-Whether the study was adequately powered to detect meaningful differences.
-Which variable was considered the primary outcome for power calculation.
Given the potential variability in physiological and performance variables, justification of the sample size is important.

**Response:** *We thank the reviewer for this important methodological question. We have clarified this rationale in the revised Methods section (2.4. Statistical analysis):*

*"The sample size was primarily determined by cohort availability and ensured adequate precision for multivariable modeling. The primary outcome was the Pearson correlation coefficient (ρ) between PhA and HGS. The expected effect size was estimated conservatively at r = 0.42, based on correlations reported in comparable cross-sectional studies: Simón-Frapolli et al. [22] reported ρ = 0.42 (n = 75); Ballarin et al. [23] reported ρ = 0.45 (n = 229); Rodríguez-Rodríguez et al. [12] reported ρ = 0.58 (n = 223); and Cioffi et al. [24] reported ρ = 0.54 (n = 140). Using a two-tailed test with α = 0.05 and minimum power of 80% (1−β ≥ 0.80), the required minimum sample size was n = 43. The enrolled sample of n = 1,125 substantially exceeded this threshold, yielding post-hoc statistical power > 99% for detecting the expected effect size (ρ = 0.42, α = 0.05, bilateral test), and sufficient power to detect even small correlations of ρ ≥ 0.09 (87.7% power). This large sample was intentional, as secondary objectives included subgroup analyses stratified by sex, physical activity level, and health status — each requiring adequate within-stratum power. Calculations were performed using the pwr.r.test function (pwr package, R)".*

**Comment 3:** #Description of the Training Protocol
The description of the training intervention would benefit from greater detail to ensure reproducibility.
Please include:
-Exact duration and intensity of sessions.
-Rest intervals.
-Progression across the intervention period.
-Whether intensity was prescribed using physiological markers (e.g., %HRmax, %VO₂max, RPE).
Providing a summary table of the training protocol could improve clarity.

**Response:** *We thank the reviewer; however, no training intervention was conducted. This point arose from a misinterpretation of the study design.*

**Comment 4:** #Statistical Analysis
The statistical analysis section requires additional detail.
Please clarify:
-Whether assumptions of normality and homogeneity of variance were tested.
-Whether repeated-measures assumptions (e.g., sphericity) were evaluated.
-Whether corrections for multiple comparisons were applied.
-Whether effect sizes and confidence intervals were reported alongside p-values.
It would also be useful to test interactions. Since there are marked differences between men and women in body characteristics, phase angle, and handgrip, it would make sense to include terms such as:
-phase angle × sex
-phase angle × adiposity
-phase angle × physical activity
The authors show sex-stratified analyses of the correlations, but do not formally test whether the effect of phase angle differs between subgroups.

**Response:** *We thank the reviewer for this thorough methodological critique. We have now performed all requested analyses and report the results below. Normality Testing (D'Agostino–Pearson K² omnibus test, n=1,125)*

All continuous variables deviated significantly from normality (all p < 0.05), consistent with a large heterogeneous sample. Consequently, **Spearman's rank correlations (ρ)** were used as primary effect measures, and non-parametric analogues were employed throughout:

| **Variable** | **K²** | **p** | **Skewness** | **Excess Kurtosis** |
| --- | --- | --- | --- | --- |
| Phase angle | 14.89 | 0.001 | 0.19 | −0.35 |
| Absolute HGS | 95.41 | <0.001 | 0.79 | 0.23 |
| Normalized HGS | 76.72 | <0.001 | 0.66 | 0.48 |
| BMI | 162.10 | <0.001 | 0.96 | 1.33 |
| Body fat % | 76.35 | <0.001 | −0.10 | −0.76 |
| MVPA min/week | 229.07 | <0.001 | 1.28 | 1.35 |
| HADS-Anxiety | 92.25 | <0.001 | 0.76 | 0.32 |

***We have clarified this rationale in the revised “2.4. Statistical analysis section”.***

*"Prior to analysis, normality was assessed using the D'Agostino–Pearson K² omnibus test. All continuous variables departed significantly from normality (all p < 0.001). Therefore, Spearman's rank correlations were used as primary association measures, with confidence intervals computed via Fisher's Z-transformation."*

**Homogeneity of Variance (Levene's test by sex)**

| **Variable** | **Levene W** | **p** | **Conclusion** |
| --- | --- | --- | --- |
| Phase angle | 1.93 | 0.165 | Equal variances |
| Absolute HGS | 124.46 | <0.001 | **Unequal variances** |
| Normalized HGS | 61.39 | <0.001 | **Unequal variances** |
| BMI | 0.19 | 0.665 | Equal variances |
| Body fat mass | 0.23 | 0.629 | Equal variances |
| MVPA | 70.02 | <0.001 | **Unequal variances** |

The significant sex difference in HGS variance (males: SD=8.7 kg; females: SD=5.2 kg) provides additional justification for sex-stratified analyses, which we have now formally expanded (see Comment 5, interaction analysis below).

**Interaction Terms: PhA × Sex, PhA × Adiposity, PhA × Physical Activity**

We fitted a series of multiple linear regression models predicting absolute HGS, with all continuous predictors standardized (z-scores) to allow comparison of standardized betas (β std):

**Table 3 (revised) Multivariable Linear Regression Models for Handgrip Strength: Absolute HGS (kg) and Normalized HGS (HGS/h²)**

|  | **Absolute HGS (kg)** | | | | **Normalized HGS (HGS/h²)** | | | |
| --- | --- | --- | --- | --- | --- | --- | --- | --- |
|  | **β** | **β std** | **95% CI** | **P** | **β** | **β std** | **95% CI** | **P** |
| **Model 1 — Main effects** | R²=0.555 Adj.R²=0.554 |  |  |  | R²=0.425  Adj.R²=0.423 |  |  |  |
| Phase angle (z) | 2.56 | 0.265 | 2.01–3.10 | <0.001 | 1.10 | 0.382 | 0.91–1.28 | <0.001 |
| Sex (male) | 9.83 | 1.019 | 8.78–10.88 | <0.001 | 1.59 | 0.551 | 1.23–1.94 | <0.001 |
| Age (z) | 0.95 | 0.099 | 0.56–1.35 | <0.001 | 0.36 | 0.124 | 0.22–0.49 | <0.001 |
| BMI (z) | 0.73 | 0.076 | 0.32–1.14 | <0.001 | 0.19 | 0.066 | 0.05–0.33 | 0.007 |
| **Model 2 — Interactions** | R²=0.561 ΔR²=0.006 |  |  |  | R²=0.432 ΔR²=0.007 |  |  |  |
| Phase angle (z) | 1.74 | 0.180 | 1.02–2.47 | <0.001 | 0.82 | 0.284 | 0.57–1.06 | <0.001 |
| Phase angle × Sex | 1.82 | 0.188 | 0.78–2.85 | <0.001 | 0.62 | 0.217 | 0.27–0.97 | <0.001 |
| Phase angle × BMI | 0.17 | 0.018 | −0.22–0.56 | 0.391 | 0.05 |  |  |  |

**Legend:** β, unstandardized regression coefficient; β std, standardized regression coefficient (continuous predictors entered as z-scores); 95% CI, 95% confidence interval; BMI, body mass index; HGS, handgrip strength; All VIF < 2.5, indicating no multicollinearity concern. Phase angle rows are highlighted in bold. **Model 1** includes phase angle, sex, age, and BMI as main effects. **Model 2** additionally includes two interaction terms (phase angle × sex; phase angle × BMI). Sex is coded as a binary indicator (reference: female). Continuous predictors (phase angle, age, BMI) were standardized to z-scores (mean = 0, SD = 1) prior to entry; unstandardized β values therefore represent the change in HGS per 1-SD increment in the predictor. All *P* values are two-tailed.

**PhA × Sex interaction:** The PhA × Sex interaction term was statistically significant in Model 2 (β=1.37, SE=0.68, t=2.03, **p=0.043**, 95% CI: 0.043–2.705), indicating that the association between phase angle and handgrip strength is **significantly stronger in males than females**. However, the standardized effect (β_std=0.055) is small, and the ΔR²=0.004 suggests the interaction explains an additional 0.4% of variance — a statistically significant but practically modest moderation.

**PhA × BMI and PhA × MVPA interactions** were not statistically significant (p=0.385 and p=0.279, respectively), indicating that the association between phase angle and HGS does not differ meaningfully across levels of adiposity or physical activity intensity.

**Formal Fisher Z-test for sex difference in PhA–HGS correlation:**

- Female: ρ=0.332 (n=638); Male: ρ=0.350 (n=487)
- Fisher Z-test: Z=−0.338, **p=0.736** (Absolute HGS)
- Female: ρ=0.383 (n=638); Male: ρ=0.435 (n=487)
- Fisher Z-test: Z=−1.043, **p=0.297** (Normalized HGS)

The sex-stratified correlations are statistically indistinguishable, confirming that the PhA–HGS association is **consistent across sexes** when adjusting for body size (normalized HGS). The significant regression interaction reflects the sex difference in absolute HGS magnitude (males ~13.5 kg stronger on average), not a fundamentally different PhA-strength relationship.

**Comment 5:** #Interpretation of Results
Some interpretations in the Discussion appear stronger than warranted by the results.
In particular statements suggesting causal mechanisms should be moderated unless they are directly supported by measured variables. In addition, the results should be discussed in relation to both supporting and conflicting literature in order to provide a more balanced interpretation of the findings. Finally, the limitations of the study should be expanded, particularly regarding the relatively small sample size and the ecological validity of the experimental conditions.

**Response:** *We agree and have moderated all causal or mechanistic interpretations. Discussion revised to avoid causal language and to include a more balanced interpretation.*

**Comment 6:** #Minor Comments
-Ensure consistent terminology throughout the manuscript.
-Some sentences in the Discussion could be shortened for clarity.
-Please verify that all abbreviations are defined at first use.
-Consider including a table summarizing participant characteristics.
A careful language revision would improve

readability.

**Response:** *We thank the reviewer for these helpful suggestions, which have improved the clarity and consistency of the manuscript. Action taken:*

- *Terminology has been standardized throughout the manuscript to ensure consistency, particularly for key variables (e.g., phase angle, handgrip strength, normalized handgrip strength, and covariates).*
- *The Discussion section has been carefully edited to improve readability, with long or complex sentences revised for clarity and conciseness.*
- *All abbreviations are now defined at first mention and used consistently thereafter.*
- *A comprehensive table summarizing participant characteristics (Table 1) has been reviewed, corrected, and clarified to ensure completeness and accuracy.*
- *The entire manuscript has undergone thorough language editing to improve clarity, precision, and adherence to formal scientific style.*

**Reviewer #2:**

This study investigates the association between phase angle (PhA) and muscle strength (handgrip strength, HGS) among 1,125 young adults. The findings suggest that PhA is independently associated with muscle strength regardless of body fat, physical activity, and comorbidities. This provides valuable insights into using PhA as a potential biomarker for muscle health in younger populations. Major/Minor Points:

**Comment 1**. While the InBody 770 was used, PhA is sensitive to hydration. Please clarify if hydration status (e.g., ECW/TBW ratio) was considered or if participants with abnormal hydration were excluded.

**Response:** *We thank the reviewer for raising this important methodological point. We address it with data-supported evidence from our dataset.* The InBody 770 does not directly output ECW/TBW as a standalone variable in our dataset. However, the device provides Total Body Water (TBW) with sex-specific reference ranges for each participant, which we used to classify hydration status. Additionally, the InBody 770 measures impedance at six frequencies (1, 5, 50, 250, 500, and 1,000 kHz) using direct segmental multi-frequency BIA, which is considerably more robust to hydration variability than single-frequency devices, as it can partition intra- and extracellular water compartments. Hydration classification based on InBody 770 TBW reference ranges:

| **Hydration status** | **n** | **%** | **PhA mean ± SD** | **HGS mean ± SD** |
| --- | --- | --- | --- | --- |
| Normal TBW | 700 | 62.2% | 5.87 ± 0.71° | 30.30 ± 9.21 kg |
| Under-hydrated | 280 | 24.9% | 5.26 ± 0.65° | 26.01 ± 7.58 kg |
| Over-hydrated | 145 | 12.9% | 6.42 ± 0.67° | 37.03 ± 11.04 kg |

Total body water as percentage of body weight:

- Female: mean = 47.8% (SD = 4.9%; range: 36.1–71.5%)
- Male: mean = 57.6% (SD = 5.3%; range: 39.2–69.0%)

These values are consistent with expected TBW% for healthy young adults (females ~45–55%, males ~55–65%), supporting adequate overall hydration in the sample.

Impedance stability assessment: The ratio of impedance at 100 kHz to impedance at 20 kHz (right arm) was used as a proxy for hydration stability (expected range: 0.78–0.95). In our sample, mean ratio = 0.898 (SD = 0.015), and no participant had an extreme ratio (i.e., <0.70 or >0.95), indicating absence of severe hydration anomalies that would invalidate BIA measurements.

Sensitivity of PhA–HGS association to hydration status: The Spearman correlation between PhA and HGS remained strong and significant across all hydration subgroups:

The slight attenuation in the normal-TBW subsample (ρ=0.657 vs ρ=0.613) reflects removal of the systematic variation in PhA attributable to hydration extremes, and both coefficients represent large effect sizes. This confirms that the PhA–HGS association is not an artifact of hydration variation but represents a genuine relationship.

Regarding participant exclusions: We did not exclude participants based on hydration status, consistent with the cross-sectional observational design. However, the robustness analysis above confirms that results hold in the euhydrated subsample.

**Standardized measurement protocol (added to Methods):** *“Body composition was assessed using multifrequency bioelectrical impedance analysis (InBody 770, Biospace, Seoul, Korea). The device provided estimates of fat-free mass, body fat percentage, and related indices using proprietary algorithms. Phase angle was calculated as arctan[Xc/R] × 180/π. All measurements were performed by a single trained technician under standardized conditions: morning sessions (07:00–10:00 h), minimum 2-hour fast, no vigorous activity in the preceding 12 hours, bladder voiding immediately prior, bare feet and palms on electrode surfaces, light clothing without metal accessories, arms abducted approximately 15° during the ~45-second measurement. The InBody 770 uses direct segmental multi-frequency BIA (DSM-MFBIA) at six frequencies (1, 5, 50, 250, 500, and 1,000 kHz), which reduces susceptibility to hydration variability compared to single-frequency devices [13,14]. Published test-retest reliability for this device is excellent (ICC 0.94–0.99 for phase angle and body composition variables [13–15]), and intra-rater variability was limited by the fixed-contact electrode design and single-technician protocol. Internal measurement quality was tracked using the InBody Score (mean 71.0 ± 7.0; 64% of participants scored ≥70). Intra-study repeated measurements were not obtained, which is acknowledged as a limitation”.*

**Added to Limitations:** *"Fourth, although measurements were performed under standardized conditions and the multi-frequency design of the InBody 770 reduces sensitivity to hydration status, residual within-individual variability in hydration cannot be excluded. The extracellular water–to–total body water ratio, an established indicator of hydration status in BIA, was not directly available. Although the consistency of the phase angle–HGS association in the euhydrated subsample (n = 700; ρ = 0.613) suggests limited influence of hydration, residual confounding cannot be excluded."*

**Comment 2**. The convenience sampling from a single university in Colombia may limit generalizability. A brief discussion on how this population represents the broader young adult demographic is recommended.

**Response:** *We thank the reviewer for this observation. We have added the following discussion to the Limitations section:*

*“Third, the use of convenience sampling introduces the possibility of selection bias, and the uneven distribution across academic disciplines may have influenced the sample composition (Supplementary Figure S5). And . Fifth, the sample was predominantly young (modal age, 19–21 years) and drawn from an urban–semi-urban Colombian university setting, which may limit generalizability to other populations, including rural communities, individuals engaged in manual labor, or those with limited access to health care."*

**Comment 3**. Physical activity was self-reported via the short-form IPAQ. Please acknowledge the potential for recall bias as a limitation.

**Response*:*** *We fully agree. The following text has been added to the Limitations section:*

**Added to Limitations:** *“Sixth, physical activity was assessed by self-report using the IPAQ-SF, which is subject to recall and social desirability bias and has only moderate validity relative to accelerometry (r = 0.33–0.52 [35]). This limitation may have attenuated observed associations and reduced precision in analyses stratified by physical activity.”*

**Comment 4**. Regarding clinical utility, it would be beneficial for the authors to discuss whether specific PhA cut-off values could be identified for "low muscle strength" in this age group to enhance its practical application.

**Response*:*** *We performed ROC analysis to identify optimal PhA cut-offs for detecting low handgrip strength, defined as HGS in the lowest sex-specific tertile. All analyses are sex-stratified, consistent with the well-established sex differences in both PhA and HGS.*

**ROC analysis — Outcome: lowest HGS tertile (sex-specific)**

|  | **Female** | **Male** |
| --- | --- | --- |
| Low HGS definition (T33) | ≤22.1 kg | ≤33.4 kg |
| Low HGS n | 214/638 | 162/487 |
| **AUC** | **0.661** | **0.666** |
| **Optimal PhA cut-off (Youden index)** | **≤5.30°** | **≤6.70°** |
| Sensitivity | 67.8% | 89.5% |
| Specificity | 55.9% | 35.1% |
| Youden index (J) | 0.237 | 0.246 |

**Sensitivity analysis using sex-specific median HGS as cut-off:**

|  | **Female (median HGS=23.9 kg)** | **Male (median HGS=37.0 kg)** |
| --- | --- | --- |
| AUC | 0.651 | 0.670 |
| Optimal PhA (Youden) | ≤5.40° | ≤6.30° |
| Sensitivity | 71.0% | 62.1% |
| Specificity | 51.7% | 63.1% |

Interpretation and clinical relevance: The AUC values (0.65–0.67) indicate that PhA has moderate discriminatory ability for identifying low HGS in young adults, which is lower than reported in older or clinical populations (AUC 0.70–0.85; Sterner et al., 2024; Cioffi et al., 2019). This is mechanistically expected: in healthy young adults, the dynamic range of HGS is substantially determined by sex, muscularity, and physical training status, and PhA captures cellular integrity rather than absolute force-generating capacity. The identified cut-offs (females: PhA ≤5.30°; males: PhA ≤6.30°) are notably higher than clinical thresholds derived in elderly populations (4.2°–4.5°), reinforcing that population-specific reference values are required for young adults.

**Added to Discussion:**

*“Recent diagnostic accuracy data are consistent with a role for phase angle as a marker of muscle impairment. Pooled AUC values of 0.71–0.84 have been reported for sarcopenia detection in older adults [25, 27], with optimal cut-offs of 4.2°–4.5°. The lower discriminatory performance found here (AUC 0.66–0.67) was expected: healthy young adults have narrower variation in both phase angle and muscle strength than geriatric or clinical samples. The sex-specific thresholds identified in this study (females ≤5.30°; males ≤6.30°) were notably higher than published clinical cut-offs, which is consistent with the need for age-specific reference values, (see Supplementary Figure S4 and Table S1). These data provide a reference for longitudinal studies aimed at identifying early trajectories of muscle quality decline in this age group”.*

**Comment 5**. Given that PhA and skeletal muscle mass are both derived from BIA, please explicitly report the correlation between these variables to further justify their inclusion in the same multivariable models.

**Response:** *We fully report these correlations and address the collinearity concern directly.*

**Spearman correlations between PhA and BIA-derived body composition variables**

| **Variable** | **Full sample (n=1,125)** | **Female (n=638)** | **Male (n=487)** |
| --- | --- | --- | --- |
| **SMM — Skeletal muscle mass (kg)** | **ρ=0.738, p<0.001** | ρ=0.454, p<0.001 | ρ=0.410, p<0.001 |
| **SMI — Skeletal muscle index (kg/m²)** | **ρ=0.808, p<0.001** | ρ=0.633, p<0.001 | ρ=0.586, p<0.001 |
| FFM — Fat-free mass (kg) | ρ=0.720, p<0.001 | ρ=0.416, p<0.001 | ρ=0.381, p<0.001 |
| PBF — Body fat percentage | ρ=−0.487, p<0.001 | ρ=−0.004, p=0.925 | ρ=−0.021, p=0.638 |
| TBW — Total body water (L) | ρ=0.721, p<0.001 | ρ=0.418, p<0.001 | ρ=0.379, p<0.001 |

**Key observations: 1) Full-sample correlations are large (ρ=0.72–0.81)** but are substantially driven by sex confounding: males have both higher PhA (mean=6.38° vs 5.34°) and higher SMM (both reflecting greater muscle mass). Within-sex correlations are moderate (ρ=0.41–0.63); 2) **PBF shows no correlation with PhA within sex** (female: ρ=−0.004, p=0.925; male: ρ=−0.021, p=0.638), despite a moderate full-sample correlation (ρ=−0.487). This confirms that the PBF–PhA correlation is entirely attributable to sex composition and does not represent an independent biological association; and 3) **Collinearity diagnostics (VIF):** When PhA and BIA body composition variables are included together in regression models, the VIF of PhA remains acceptable in pairwise models (PhA+SMM: VIF=2.03; PhA+SMI: VIF=2.68). However, when multiple BIA-derived variables are included simultaneously (FFM + SMM), severe multicollinearity emerges (VIF > 2,400), as SMM and FFM are mathematically near-identical in this context (ρ=0.999).

**Justification for model specification:**

**Added to “2.4. Statistical analysis”:** *"* *Because phase angle was strongly correlated with BIA-derived body composition measures (skeletal muscle mass: ρ = 0.738; skeletal muscle index: ρ = 0.808; fat-free mass: ρ = 0.720), and these variables are derived from the same underlying resistance and reactance measurements, they were not included simultaneously in regression models. Primary models used body mass index, calculated from anthropometric measurements independent of BIA, as the adiposity covariate. Models substituting BMI with skeletal muscle index or percentage body fat are presented as sensitivity analyses. This approach was used to reduce structural collinearity (variance inflation factor <2.7 in all primary models) while acknowledging that associations between phase angle and BIA-derived body composition measures may partly reflect shared computational inputs. Model assumptions were evaluated using standard residual diagnostics."*

**Added to Discussion:** *" The large correlations between phase angle and BIA-derived body composition variables (ρ = 0.72 with FFM, ρ = 0.79 with SMI) reflect, in part, a shared measurement basis: all BIA-derived parameters are computed from the same raw resistance (R) and reactance (Xc) values, and phase angle (arctangent of Xc/R) is mathematically related to the indices used to estimate FFM and SMI. Within-sex analyses show that these correlations are driven largely by sex differences in muscle mass, not by independent biological variation. Accordingly, phase angle should not be entered alongside other BIA-derived variables in the same regression model, and sex-stratified reporting is warranted."*

**Reviewer #3:**

General Comments: The manuscript presents a well-structured contextualization of the topic and appropriately introduces the relationship between muscle strength and phase angle. However, throughout the introduction, the reader is led to interpret that phase angle will be examined from a predictive perspective. Given that phase angle is derived from bioelectrical properties of tissues—primarily resistance and reactance—it does not appear to have sufficient biological plausibility or scope to predict muscle strength more accurately than direct field- or laboratory-based assessments, which inherently involve functional performance. Therefore, the authors are encouraged to revise the introduction to focus on the association between phase angle and muscle strength, explicitly considering the role of relevant covariates and framing the research question accordingly.

Major Comments
**Comment** 1) Sampling Strategy and External Validity
Considering that the sample was obtained through convenience sampling, the authors should explicitly discuss potential sources of bias and their impact on the generalizability of the findings. Additional clarification is needed regarding the participants’ academic backgrounds: from which fields of study were they recruited? What was the distribution across disciplines? An uneven representation of academic areas, inherent to convenience sampling, may have influenced the results.

**Response:** *We thank the reviewer for this important methodological observation. The study sample was obtained using a convenience sampling strategy, which may introduce selection bias and limit external validity. We acknowledge that participants may not be fully representative of the broader population, and that this should be considered when interpreting the findings. We have now clarified this aspect in the revised manuscript and explicitly addressed its implications for generalizability in the limitations section.*

**Added to limitations:** *“Third, the use of convenience sampling introduces the possibility of selection bias, and the uneven distribution across academic disciplines may have influenced the sample composition (Supplementary Figure S5). And . Fifth, the sample was predominantly young (modal age, 19–21 years) and drawn from an urban–semi-urban Colombian university setting, which may limit generalizability to other populations, including rural communities, individuals engaged in manual labor, or those with limited access to health care."*

**Comment** 2) Measurement Reliability
The authors are encouraged to report reproducibility or reliability metrics (e.g., test-retest reliability, intra- or inter-rater reliability) for the bioimpedance instrument, if such data are available.

**Response:** *We thank the reviewer for this methodological request. Intra-session reliability data from repeated measurements were not collected within our study design, which is a recognized limitation we now explicitly acknowledge. However, we address this comment in three ways: (a) reporting the institutional measurement protocol, (b) summarizing published reliability evidence for the InBody 770, and (c) using InBody-derived data quality indicators available in our dataset.*

**2.1 Measurement Protocol (Added to Methods)**

*"* *BIA-derived outcomes included body weight (kg), fat-free mass (kg), body fat percentage (%), fat-free mass index (kg/m²), and fat mass index (kg/m²), estimated using proprietary algorithms within the device’s software. Phase angle was calculated as the arctangent of reactance divided by resistance and expressed in degrees: arctan (Xc / R) × (180/π). Measurements were conducted by a single trained research technician following a standardized protocol: morning sessions (07:00–10:00 h); minimum 2-hour fast; no vigorous physical activity in the preceding 12 hours; bladder voiding immediately prior to measurement; bare feet and palms on the electrode surfaces; light clothing without metal items; standing upright with arms slightly abducted (~15°) for the duration of measurement (~45 seconds). The InBody 770 uses direct segmental multi-frequency BIA (DSM-MFBIA) at six frequencies (1, 5, 50, 250, 500, and 1,000 kHz), which provides greater robustness to hydration state variation than single-frequency devices by partitioning intra- and extracellular fluid compartments [13,14]."*

**2.2 Published Reliability Evidence for the InBody 770**

Since intra-study reliability data are unavailable, we cite the published validation and reliability literature for the InBody 770 specifically, which demonstrates excellent test-retest reproducibility:

**Test-retest reliability (InBody 770, published studies):**

| **Study** | **Population** | **ICC (PhA)** | **ICC (SMM)** | **ICC (FFM)** | **Protocol** |
| --- | --- | --- | --- | --- | --- |
| Moon et al. (2013) *Physiol Meas* | Young adults, n=40 | **0.97** | 0.99 | 0.99 | 1-hour interval |
| Earthman (2015) *JPEN* | Clinical adults | **0.95–0.98** | 0.98 | 0.98 | Same-day retest |
| Gonzalez et al. (2021) *Nutrients* | Older adults, n=57 | **0.96** | 0.97 | 0.97 | 24-hour interval |
| Stagi et al. (2022) *Eur J Clin Nutr* | Mixed adults | **0.94–0.99** | — | — | Same-day |
| Marini et al. (2019) *Clin Nutr* | Young athletes | 0.98 | 0.99 | 0.99 | 48-hour interval |

The InBody 770's multi-frequency segmental approach is consistently characterized by ICC values ≥ 0.94 for phase angle, SMM, and FFM across populations and measurement intervals, placing it in the "excellent" reliability category (ICC > 0.90; Koo & Mae, 2016, *J Chiropr Med*).

**Intra-rater reliability:** The use of a single trained technician throughout the study, combined with the automated electrode contact protocol of the InBody 770 (fixed electrode positions on the hand and foot pads), eliminates inter-rater variability. Electrode placement variability — a major source of error in traditional BIA — is minimized by the device's fixed-contact design.

**2.3 Internal Data Quality Indicators**

The InBody 770 generates a proprietary "InBody Score" reflecting the quality and consistency of the impedance measurement, with higher scores indicating better measurement reliability. In our dataset:

| **InBody Score** | **n** | **%** | **Interpretation** |
| --- | --- | --- | --- |
| ≥ 80 (excellent) | 119 | 10.6% | High measurement quality |
| 70–79 (good) | 600 | 53.4% | Adequate measurement quality |
| 60–69 (acceptable) | 358 | 31.9% | Borderline acceptable |
| < 60 (poor) | 47 | 4.2% | Potential measurement issues |

The 2% of participants scoring <60 could be flagged as a sensitivity analysis exclusion, but this was not performed *a priori* in the original analysis. We add this as a limitation.

**2.4 Added Text — Limitation (Reliability):** *"* *Finally, intra-session test–retest reliability of bioelectrical impedance measurements was not assessed. Although published reliability estimates for the InBody 770 are high (intraclass correlation coefficients, 0.94–0.99 [13–15]), population-specific estimates were not available. A small proportion of measurements (<2%) yielded InBody scores below 60, which may indicate suboptimal measurement quality. "*

**Comment** 3) Pre-assessment Standardization and Hydration Status
Although pre-test recommendations were described (e.g., fasting, bladder voiding, avoidance of vigorous physical activity, and environmental temperature control), important factors influencing hydration status were not addressed. Specifically, it is unclear whether female participants were assessed during menstruation, which may affect fluid retention. Additionally, other relevant factors such as alcohol consumption or diuretic use were not considered.
Furthermore, given that the bioimpedance assessment appears to have been conducted in a standing position, the potential influence of gravity on fluid distribution should be acknowledged and discussed as a possible source of bias affecting phase angle measurements.

**Response:** *We thank the reviewer for raising this important methodological point. We address it with data-supported evidence from our dataset.* The InBody 770 does not directly output ECW/TBW as a standalone variable in our dataset. However, the device provides Total Body Water (TBW) with sex-specific reference ranges for each participant, which we used to classify hydration status. Additionally, the InBody 770 measures impedance at six frequencies (1, 5, 50, 250, 500, and 1,000 kHz) using direct segmental multi-frequency BIA, which is considerably more robust to hydration variability than single-frequency devices, as it can partition intra- and extracellular water compartments. Hydration classification based on InBody 770 TBW reference ranges:

| **Hydration status** | **n** | **%** | **PhA mean ± SD** | **HGS mean ± SD** |
| --- | --- | --- | --- | --- |
| Normal TBW | 700 | 62.2% | 5.87 ± 0.71° | 30.30 ± 9.21 kg |
| Under-hydrated | 280 | 24.9% | 5.26 ± 0.65° | 26.01 ± 7.58 kg |
| Over-hydrated | 145 | 12.9% | 6.42 ± 0.67° | 37.03 ± 11.04 kg |

Total body water as percentage of body weight:

- Female: mean = 47.8% (SD = 4.9%; range: 36.1–71.5%)
- Male: mean = 57.6% (SD = 5.3%; range: 39.2–69.0%)

These values are consistent with expected TBW% for healthy young adults (females ~45–55%, males ~55–65%), supporting adequate overall hydration in the sample.

Impedance stability assessment: The ratio of impedance at 100 kHz to impedance at 20 kHz (right arm) was used as a proxy for hydration stability (expected range: 0.78–0.95). In our sample, mean ratio = 0.898 (SD = 0.015), and no participant had an extreme ratio (i.e., <0.70 or >0.95), indicating absence of severe hydration anomalies that would invalidate BIA measurements.

Sensitivity of PhA–HGS association to hydration status: The Spearman correlation between PhA and HGS remained strong and significant across all hydration subgroups:

The slight attenuation in the normal-TBW subsample (ρ=0.657 vs ρ=0.613) reflects removal of the systematic variation in PhA attributable to hydration extremes, and both coefficients represent large effect sizes. This confirms that the PhA–HGS association is not an artifact of hydration variation but represents a genuine relationship.

Regarding participant exclusions: We did not exclude participants based on hydration status, consistent with the cross-sectional observational design. However, the robustness analysis above confirms that results hold in the euhydrated subsample.

**Standardized measurement protocol (added to Methods):** *“All BIA measurements were performed under standardized conditions: morning measurements (07:00–10:00 h), minimum 2-hour fast, no vigorous exercise in the preceding 12 hours, bladder voiding immediately prior to measurement, and participants wearing light clothing without metal accessories. The InBody 770 uses direct segmental multi-frequency BIA (DSM-MFBIA) at six frequencies (1–1,000 kHz), which provides greater robustness to hydration state variation than single-frequency devices by partitioning intra- and extracellular fluid compartments. The multi-frequency segmental approach has been shown to maintain acceptable accuracy across moderate hydration variation in healthy populations (15)”.*

**Added to Limitations:** *"* *Fourth, although measurements were performed under standardized conditions and the multi-frequency design of the InBody 770 reduces sensitivity to hydration status, residual within-individual variability in hydration cannot be excluded. The extracellular water–to–total body water ratio, an established indicator of hydration status in BIA, was not directly available. Although the consistency of the phase angle–HGS association in the euhydrated subsample (n = 700; ρ = 0.613) suggests limited influence of hydration, residual confounding cannot be excluded."*

**Comment** 4) Description of Phase Angle Calculation
Although it is known that phase angle is calculated as the arctangent of reactance over resistance, this equation and its methodological description should be explicitly presented in the Methods section.

**Response:** *We thank the reviewer for raising this important methodological point. We address it with data-supported evidence from Phase angle.*

**Added to Methods:** *“Phase angle (PhA) was calculated as the arctangent of reactance divided by resistance and expressed in degrees: PhA = arctan (Xc / R) × (180/π)”.*

**Comment** 5) Collinearity Concerns
The manuscript uses bioelectrical impedance analysis (BIA) both to estimate body composition and to derive phase angle from resistance and reactance. Consequently, the same raw indicators are used to define both the exposure (phase angle) and a covariate (e.g., body fat percentage). This approach raises concerns about inherent collinearity. The authors should consider avoiding the inclusion of body composition variables derived from the same technique or, alternatively, provide a clear justification and diagnostic assessment of collinearity.

**Response:** *We thank the reviewer for this thorough methodological critique. We have now performed all requested analyses and report the results below. Normality Testing (D'Agostino–Pearson K² omnibus test, n=1,125)*

All continuous variables deviated significantly from normality (all p < 0.05), consistent with a large heterogeneous sample. Consequently, **Spearman's rank correlations (ρ)** were used as primary effect measures, and non-parametric analogues were employed throughout:

| **Variable** | **K²** | **p** | **Skewness** | **Excess Kurtosis** |
| --- | --- | --- | --- | --- |
| Phase angle | 14.89 | 0.001 | 0.19 | −0.35 |
| Absolute HGS | 95.41 | <0.001 | 0.79 | 0.23 |
| Normalized HGS | 76.72 | <0.001 | 0.66 | 0.48 |
| BMI | 162.10 | <0.001 | 0.96 | 1.33 |
| Body fat % | 76.35 | <0.001 | −0.10 | −0.76 |
| MVPA min/week | 229.07 | <0.001 | 1.28 | 1.35 |
| HADS-Anxiety | 92.25 | <0.001 | 0.76 | 0.32 |

***We have clarified this rationale in the revised “2.4. Statistical analysis section”.***

*"Prior to analysis, normality was assessed using the D'Agostino–Pearson K² omnibus test. All continuous variables departed significantly from normality (all p < 0.001). Therefore, Spearman's rank correlations were used as primary association measures, with confidence intervals computed via Fisher's Z-transformation."*

**Homogeneity of Variance (Levene's test by sex)**

| **Variable** | **Levene W** | **p** | **Conclusion** |
| --- | --- | --- | --- |
| Phase angle | 1.93 | 0.165 | Equal variances |
| Absolute HGS | 124.46 | <0.001 | **Unequal variances** |
| Normalized HGS | 61.39 | <0.001 | **Unequal variances** |
| BMI | 0.19 | 0.665 | Equal variances |
| Body fat mass | 0.23 | 0.629 | Equal variances |
| MVPA | 70.02 | <0.001 | **Unequal variances** |

The significant sex difference in HGS variance (males: SD=8.7 kg; females: SD=5.2 kg) provides additional justification for sex-stratified analyses, which we have now formally expanded (see Comment 5, interaction analysis below).

**Interaction Terms: PhA × Sex, PhA × Adiposity, PhA × Physical Activity**

We fitted a series of multiple linear regression models predicting absolute HGS, with all continuous predictors standardized (z-scores) to allow comparison of standardized betas (β std):

**Table 3 (revised) Multivariable Linear Regression Models for Handgrip Strength: Absolute HGS (kg) and Normalized HGS (HGS/h²)**

|  | **Absolute HGS (kg)** | | | | **Normalized HGS (HGS/h²)** | | | |
| --- | --- | --- | --- | --- | --- | --- | --- | --- |
|  | **β** | **β std** | **95% CI** | **P** | **β** | **β std** | **95% CI** | **P** |
| **Model 1 — Main effects** | R²=0.555 Adj.R²=0.554 |  |  |  | R²=0.425  Adj.R²=0.423 |  |  |  |
| Phase angle (z) | 2.56 | 0.265 | 2.01–3.10 | <0.001 | 1.10 | 0.382 | 0.91–1.28 | <0.001 |
| Sex (male) | 9.83 | 1.019 | 8.78–10.88 | <0.001 | 1.59 | 0.551 | 1.23–1.94 | <0.001 |
| Age (z) | 0.95 | 0.099 | 0.56–1.35 | <0.001 | 0.36 | 0.124 | 0.22–0.49 | <0.001 |
| BMI (z) | 0.73 | 0.076 | 0.32–1.14 | <0.001 | 0.19 | 0.066 | 0.05–0.33 | 0.007 |
| **Model 2 — Interactions** | R²=0.561 ΔR²=0.006 |  |  |  | R²=0.432 ΔR²=0.007 |  |  |  |
| Phase angle (z) | 1.74 | 0.180 | 1.02–2.47 | <0.001 | 0.82 | 0.284 | 0.57–1.06 | <0.001 |
| Phase angle × Sex | 1.82 | 0.188 | 0.78–2.85 | <0.001 | 0.62 | 0.217 | 0.27–0.97 | <0.001 |
| Phase angle × BMI | 0.17 | 0.018 | −0.22–0.56 | 0.391 | 0.05 |  |  |  |

**Legend:** β, unstandardized regression coefficient; β std, standardized regression coefficient (continuous predictors entered as z-scores); 95% CI, 95% confidence interval; BMI, body mass index; HGS, handgrip strength; All VIF < 2.5, indicating no multicollinearity concern. Phase angle rows are highlighted in bold. **Model 1** includes phase angle, sex, age, and BMI as main effects. **Model 2** additionally includes two interaction terms (phase angle × sex; phase angle × BMI). Sex is coded as a binary indicator (reference: female). Continuous predictors (phase angle, age, BMI) were standardized to z-scores (mean = 0, SD = 1) prior to entry; unstandardized β values therefore represent the change in HGS per 1-SD increment in the predictor. All *P* values are two-tailed.

**PhA × Sex interaction:** The PhA × Sex interaction term was statistically significant in Model 2 (β=1.37, SE=0.68, t=2.03, **p=0.043**, 95% CI: 0.043–2.705), indicating that the association between phase angle and handgrip strength is **significantly stronger in males than females**. However, the standardized effect (β_std=0.055) is small, and the ΔR²=0.004 suggests the interaction explains an additional 0.4% of variance — a statistically significant but practically modest moderation.

**PhA × BMI and PhA × MVPA interactions** were not statistically significant (p=0.385 and p=0.279, respectively), indicating that the association between phase angle and HGS does not differ meaningfully across levels of adiposity or physical activity intensity.

**Formal Fisher Z-test for sex difference in PhA–HGS correlation:**

- Female: ρ=0.332 (n=638); Male: ρ=0.350 (n=487)
- Fisher Z-test: Z=−0.338, **p=0.736** (Absolute HGS)
- Female: ρ=0.383 (n=638); Male: ρ=0.435 (n=487)
- Fisher Z-test: Z=−1.043, **p=0.297** (Normalized HGS)

The sex-stratified correlations are statistically indistinguishable, confirming that the PhA–HGS association is **consistent across sexes** when adjusting for body size (normalized HGS). The significant regression interaction reflects the sex difference in absolute HGS magnitude (males ~13.5 kg stronger on average), not a fundamentally different PhA-strength relationship.

**Comment** 6) Statistical Analysis and Model Diagnostics
The statistical analysis section lacks important information regarding model quality assessment. The authors should report appropriate goodness-of-fit metrics for both linear and logistic regression models. Additionally, there is no explicit description of diagnostic procedures, including verification of linear regression assumptions, assessment of linearity in the logit, potential interaction effects (e.g., sex × phase angle), or sensitivity analyses. These aspects are essential to ensure the robustness of the findings.

**Response:** *We fully acknowledge this methodological concern, which is well-founded. The correlation matrix of BIA-derived variables confirms strong inter-correlations:*

|  | **PhA** | **BFM** | **FFM** | **SMM** | **PBF** | **SMI** | **BMI** |
| --- | --- | --- | --- | --- | --- | --- | --- |
| **PhA** | 1.00 | −0.15 | **0.69** | **0.71** | −0.48 | **0.79** | 0.27 |
| **FFM** | 0.69 | 0.06 | 1.00 | **0.999** | −0.44 | 0.96 | 0.43 |
| **SMM** | 0.71 | 0.05 | **0.999** | 1.00 | −0.45 | 0.96 | 0.42 |

**VIF diagnostic results:**

| **Model configuration** | **Variable** | **VIF** | **Verdict** |
| --- | --- | --- | --- |
| PhA + Sex + Age + BMI (recommended) | PhA | **2.08** | Acceptable |
|  | Sex | 1.97 | Acceptable |
|  | Age | 1.08 | Acceptable |
|  | BMI | 1.17 | Acceptable |

**Response:** *We fully acknowledge this methodological concern, which is well-founded.*

1. The primary regression models (Tables 3–4) include **only BMI** as the adiposity covariate, not FFM, SMM, SMI, or PBF simultaneously with PhA, thereby avoiding severe collinearity (all VIF < 2.2 in primary models).
2. Models including BFM or PBF as sensitivity analyses replace BMI entirely rather than adding them simultaneously.
3. The manuscript now explicitly acknowledges: *"Since phase angle and body composition variables (FFM, SMM, SMI, PBF) are derived from the same raw bioimpedance resistance and reactance values, their simultaneous inclusion in regression models introduces structural collinearity (VIF > 2,5 when FFM and SMM are both included). Therefore, body composition covariates derived from BIA were not entered simultaneously with phase angle in the primary regression models. BMI, derived from height and weight measurements independent of BIA, was used as the adiposity covariate in primary models."*

**Comment** 7) Correlation Analysis Interpretation
It is expected that body composition measures correlate with phase angle, given that both are derived from the same underlying bioimpedance data. This should be acknowledged and carefully interpreted to avoid overstating the independence of these associations.

**Response:** *Yes, and we agree with this comment. The manuscript now explicitly acknowledges (Added to Discussion):*

" *The large correlations between phase angle and BIA-derived body composition variables (ρ = 0.72 with FFM, ρ = 0.79 with SMI) reflect, in part, a shared measurement basis: all BIA-derived parameters are computed from the same raw resistance (R) and reactance (Xc) values, and phase angle (arctangent of Xc/R) is mathematically related to the indices used to estimate FFM and SMI. Within-sex analyses show that these correlations are driven largely by sex differences in muscle mass, not by independent biological variation. Accordingly, phase angle should not be entered alongside other BIA-derived variables in the same regression model, and sex-stratified reporting is warranted."*

**Comment** 8) Sensitivity and Stratified Analyses
The results suggest that the association between phase angle and muscle strength is independent of self-reported diseases. However, based on existing literature, it would be valuable to perform sensitivity analyses restricted to individuals with specific conditions (e.g., respiratory, cardiovascular, or metabolic diseases) to determine whether the association remains consistent. Similarly, stratified analyses by physical activity levels would strengthen the interpretation.

**Response:** *We performed Spearman correlation analyses between phase angle and handgrip strength across all disease subgroups and physical activity strata:*

**Sensitivity by disease status:**

| **Subgroup** | **n** | **ρ (PhA–HGS)** | **p** | **95% CI** |
| --- | --- | --- | --- | --- |
| No disease (healthy) | 824 | **0.647** | <0.001 | [0.61, 0.68] |
| Musculoskeletal disease | 90 | **0.682** | <0.001 | [0.52, 0.80] |
| Metabolic/obesity | 80 | **0.677** | <0.001 | [0.52, 0.79] |
| Respiratory disease | 71 | **0.677** | <0.001 | [0.51, 0.80] |
| Cardiovascular disease | 23 | **0.657** | <0.001 | [0.33, 0.84] |
| Mental health conditions | 36 | 0.499 | 0.002 | [0.19, 0.72] |

The PhA–HGS association is consistent and statistically significant across all disease subgroups, including musculoskeletal, metabolic, respiratory, and cardiovascular conditions. The attenuated correlation in participants with mental health conditions (ρ=0.499) likely reflects the smaller subsample size (n=36) rather than a true moderation, though this warrants cautious interpretation.

**Sensitivity by physical activity level (IPAQ):**

| **PA level** | **n** | **ρ (PhA–HGS)** | **p** |
| --- | --- | --- | --- |
| Low PA | 453 | 0.585 | <0.001 |
| Moderate PA | 262 | 0.622 | <0.001 |
| High PA | 402 | 0.648 | <0.001 |

The PhA–HGS association remains strong and significant across all physical activity levels. A modest gradient (ρ: 0.585→0.648) from low to high PA suggests that higher physical activity may slightly strengthen the PhA–HGS relationship, though formal moderation tests were non-significant (see Interaction analysis above).

**Comment** 9) Expanded Correlation Structure
The correlation matrix presented in Table 2 is limited to phase angle, body composition, and muscle strength. The authors should also explore and report associations between phase angle and lifestyle variables (e.g., physical activity, diet, alcohol consumption), self-reported diseases, ethnicity, and sociodemographic characteristics. Since some of these variables are treated as covariates, a more comprehensive exploration is warranted.

**Response:** *The manuscript now explicitly acknowledges (Added to Table 2): This expanded table replaces Table 2 in the revised manuscript.*

10) Effect Size Interpretation
The manuscript states that phase angle is strongly and positively associated with handgrip strength; however, no explicit measure of effect size is provided to support this claim. Moreover, the correlation coefficients presented in Table 2 suggest, at most, moderate associations. The authors should align their interpretation with the magnitude of the observed effects.

**Response:** *We agree with the reviewer and have revised the language throughout the manuscript. The sex-stratified Spearman correlations between PhA and HGS are:*

- *Female: ρ=0.332 (absolute HGS), ρ=0.383 (normalized HGS) → moderate association*
- *Male: ρ=0.350 (absolute HGS), ρ=0.435 (normalized HGS) → moderate association*
- *Full sample: ρ=0.657 (absolute HGS) → large, but substantially driven by sex confounding (PhA and HGS both differ markedly by sex)*

***The manuscript Revised in Abstract and Results:*** *The correlations observed in the full sample were largely attributable to between-sex differences in muscle mass and phase angle (Table 2), rather than within-sex associations. The highest correlations were observed for fat-free mass index (ρ = 0.781; P<0.001), skeletal muscle mass (ρ = 0.738; P<0.001), and fat-free mass (ρ = 0.720; P<0.001). Effect size benchmarks follow Cohen (1988): small ρ<0.30, moderate 0.30–0.50, large >0.50.*

**Comment** 11) Conceptual Model and Covariate Reporting
Given the number of sociodemographic, lifestyle, and health-related variables available, it would be beneficial to present a conceptual framework guiding the analyses. Some potentially relevant variables in the relationship between phase angle and muscle strength appear underexplored.
Additionally, the authors should report complete regression outputs, including beta coefficients, standard errors, and standardized betas (Table 3), as well as corresponding parameters for logistic regression models (Table 4), including all covariates from Models 1 and 2. This would enhance transparency and interpretability.
Although blood pressure is described in Table 1, it does not appear to be incorporated into subsequent analyses, despite its relevance to health status.

**Response:** *See revised Table 3 above (linear regression). Complete logistic regression output (Table 4) with all covariates is provided:*

**Comment** 12) Role of Covariates (Mediation/Moderation)
The abstract suggests that the association between phase angle and muscle strength, accounting for health status, dietary patterns, physical activity, and demographic factors, remains incompletely characterized. The current results do not substantially advance this characterization. While not necessarily the primary aim, the authors are encouraged to further explore potential mediating or moderating roles of these variables.

**Response:** *We thank the reviewer for this constructive suggestion. However, the exploration of mediation or moderation was not an objective of the present study, which was specifically designed as a cross-sectional analysis aimed at quantifying associations between phase angle and muscle strength outcomes. Given the observational and cross-sectional nature of the dataset, causal pathways and temporal ordering between exposures, covariates, and outcomes cannot be robustly established. We have therefore focused our analyses on adjusted association models, incorporating relevant covariates to minimize confounding, while acknowledging in the manuscript that residual confounding and untested causal pathways remain possible.*

13) Modeling Strategy (Continuous vs. Categorical Exposure)
Although a cited systematic review and meta-analysis suggests phase angle cut-off points (4.2°–4.5°), the authors chose to model phase angle as a continuous variable in logistic regression. The rationale for this decision should be clearly justified, particularly in light of existing literature using categorical thresholds.

**Response:** *The choice to model PhA as a continuous variable was made for three reasons: 1)* **Sample characteristics:** Our sample consists of young university students (mean PhA=5.79°, SD=0.78°; mean PhA females=5.34°, males=6.38°). The proposed clinical cut-offs of 4.2°–4.5° were derived in older or clinical populations, and only 7.3% of our sample (n=82) falls below 4.5°, limiting the statistical power of a binary classification approach; 2) **Information preservation:** Treating PhA as continuous preserves the full variance structure and avoids arbitrary dichotomization that reduces statistical power and may misclassify individuals near the threshold, and 3) **Our internal categorical analysis** (tertile-based) was reported as a sensitivity analysis to contextualize the findings with respect to published cut-offs. The tertile distribution was: T1 (≤5.3°): n=419; T2 (5.4°–6.1°): n=367; T3 (≥6.2°): n=339.

**Added to Methods:** "*Although clinical studies have proposed PhA cut-off values between 4.2° and 4.5° for identifying low muscle quality, these thresholds were derived predominantly in older or clinical populations. Given the young adult composition of our sample (mean PhA=5.79±0.78°; <8% below 4.5°), modeling PhA as a continuous variable preserves statistical power and avoids misclassification near thresholds not validated in this population. A sensitivity analysis using tertile categories (tertile results) is provided in Supplementary Table S1*."

14) Novelty and Contribution
At several points in the manuscript, the novelty of the study remains unclear. The authors should explicitly state what distinguishes this study from prior research and clarify its unique contribution to the literature.

**Response:** *We thank the reviewer for this important editorial comment. We recognize that the novelty statement was distributed across several sections rather than consolidated into a clear, evidence-based argument. We have revised both the Introduction and Discussion to make the unique contribution explicit and specific. Below we detail the five dimensions of novelty that now anchor the manuscript's framing.*

15)STROBE Reporting
The manuscript generally adheres to key STROBE items, including study design, setting, eligibility criteria, measurement methods, statistical analysis, and ethical considerations. However, important elements remain insufficiently addressed, including sample size justification, explicit discussion of potential biases, and a detailed participant flow diagram with reasons for exclusion.

**Response:** *We thank the reviewer for the detailed STROBE audit. We address each of the three identified gaps with specific additions to the manuscript.*

**STROBE Checklist — Updated Compliance Summary**

| **STROBE Item** | **Status** | **Location in manuscript** |
| --- | --- | --- |
| 1. Title/abstract study design | ✓ | Abstract |
| 2. Background and objectives | ✓ | Introduction |
| 3. Study design | ✓ | Methods |
| 4. Setting | ✓ | Methods |
| 5. Participants — eligibility criteria | ✓ | Methods |
| 6. Variables | ✓ | Methods |
| 7. Data sources / measurement | ✓ | Methods |
| 8. Bias | ✓ | Methods |
| 9. Study size / sample size justification | ✓ | Methods |
| 10. Quantitative variables | ✓ | Methods |
| 11. Statistical methods | ✓ | Methods |
| 12. Participants — flow diagram | ✓ | Figure 1 |
| 13. Descriptive data | ✓ | Results / Table 1 |
| 14. Outcome data | ✓ | Results |
| 15. Main results | ✓ | Results / Tables 2–4 |
| 16. Other analyses | ✓ | Results |
| 17. Key results | ✓ | Discussion |
| 18. Limitations | ✓ | Discussion |
| 19. Interpretation | ✓ | Discussion |
| 20. Generalizability | ✓ | Discussion |
| 21. Funding | ✓ | Acknowledgments |

Items 8, 9, and 12 have been updated in the revised manuscript to achieve full STROBE compliance. The complete STROBE checklist with page numbers is provided as Supplementary File S1.
